# Sociodemographic characteristics, social support, and family history as factors of depression, anxiety, and stress among young adult senior high school students in metro Manila, Philippines, during the COVID-19 pandemic

**DOI:** 10.3389/fpsyt.2023.1225035

**Published:** 2023-09-13

**Authors:** Ian Marie Atasha Serrano, Anne Marie Nicole Cuyugan, Krischel Cruz, Jan Mae Ann Mahusay, Rowalt Alibudbud

**Affiliations:** Department of Sociology and Behavioral Sciences, De La Salle University, Manila, Philippines

**Keywords:** Philippines, student mental health, school mental health, depression, anxiety, stress, students, social support

## Abstract

**Introduction:**

Academic institutions must consider the students at risk for developing poor mental health and the factors influencing them. With the scarcity of literature concerning student mental health in the Philippines, this study determined the factors influencing the risk of anxiety, depression, and stress among senior high school students in metro Manila.

**Methods:**

This cross-sectional study explored the influence of sociodemographic characteristics, social support, and family history on depression, anxiety, and stress among young adult senior high school students using the Depression, Anxiety, and Stress Scale-21 (DASS-21) and the Multidimensional Scale of Perceived Social Support (MSPSS). After describing the data, regression analyses were performed.

**Results:**

A total of 187 participants were recruited. Three out of five participants have a significant risk for depression. Four out of five participants were at significant risk for anxiety. One out of four participants was at risk for significant stress. Social support from a significant other and one’s family may be protective factors for significant depression among the participants. Having female sex may be a risk factor for significant anxiety. In addition, having female sex and a family history of mental disorders may be risk factors for significant stress.

**Discussion:**

Social support should be strengthened among senior high school students to improve their mental well-being. Students at risk for poor mental health, including females and those with a family history of mental disorders, may need additional support in school mental health programs. Finally, further research is recommended to fully understand mental health among Filipino students.

## 1. Introduction

Previous studies have noted that young adult students had an increase in the severity of mental health problems, such as higher levels of significant depression, anxiety, and stress symptoms, during the COVID-19 pandemic (1, 2). Moreover, as the pandemic progressed, students continued to suffer from mental health problems (2). If neglected, mental health problems may have a detrimental impact on a student’s physical health, academic performance, and overall ability to work long-term; (3). As a result, it is crucial for academic institutions to take into account the students at risk for developing mental health problems such as depression, anxiety, and stress and to take note of the factors influencing them.

### 1.1. Mental health problems among students in the Philippines

The Philippines’ mental health burden has increased, with 35.89% of the Filipino population exhibiting moderate to severe anxiety symptoms during the COVID-19 pandemic (4). Recent publications, such as the Philippine Development Plan 2017–2023, have emphasized that young adults are among society’s most vulnerable population groups, as childhood is a critical phase in which these illnesses emerge (5). Moreover, it is estimated that 16% of children had a mental disorder prior to the pandemic (5). Likewise, another local study in a single institution setting estimated that about 35 and 47.2% of students are at risk for depressive and anxiety disorders (6). During the pandemic, the mental health burden seemingly worsened, with suicide attempts among Filipino youth increasing to 7.5% in 2021 from 3% prior to the pandemic (7). Therefore, it is essential to assess the factors contributing to the mental health burden of Filipino youth during the pandemic.

According to the 2021 Young Adult Fertility and Sexuality Study (YAFS5), a fifth of the population of “Metro Manila” or National Capital Region (NCR), one of the Philippines’ most populous region, consist of young people aged 15–24 or about 2.4 million people (8) Among them, 51.7% are females and 48.3% are males, while 26.4% are senior high school students taking grades 11 and 12 (8). Moreover, this number of young people accounts for about 12% of the Philippines’ young adult population (8). Therefore, with a relatively large portion of the Filipino youth, the factors influencing the mental health of young people from Metro Manila can be determined to address and reduce their magnified mental health burden during the COVID-19 pandemic.

In a similar context, Labasano discovered that one out of every two students suffered from severe anxiety symptoms in a senior high school setting (9). Another study showed a significant relationship between anxiety and certain influential factors, namely, sociodemographic and psychological factors (10). While there is undoubtedly an alarming increase in students experiencing mental health issues, Cleofas observed that data on anxiety among students in the Philippines remains elusive (11). AlKandari stated that these services include efficient mental guidance with the help of workers who specialize in psychology and counseling (12). Furthermore, AlKandari noted that these services give students a safe, secure, and open environment they can seek in times of difficulty (12). However, to effectively establish these services, it is necessary to fully recognize the critical factors essential to understanding and determining the needs of these students (11). Given the heightened mental health burden among young people in the Philippines and the need and elusiveness of data regarding Filipino students, it is, therefore, necessary to determine the rate and factors influencing the mental health of young Filipino students.

### 1.2. Factors affecting student mental health

Various factors influence anxiety, depression, and stress among students. These include sociodemographic characteristics, biological factors, and social factors. For instance, age, sex assigned at birth, sexual orientation, household income level, religious affiliation, family history of mental health disorders, and social support may influence anxiety (13–15).

Ultimately, 14% of adolescents aged 10 to 19 experience mental health distress (16). Moreover, there was consistent evidence that female adolescents revealed more anxiety concerns than males (14). This was further supported by the study of Gao Wenjuan et al. claiming that a large proportion of female students experience anxiety, while a higher percentage of male students suffer from depression (17). Another study in the Philippines explored sexual orientation and mental health, indicating that sexual minority women have higher rates of depression, anxiety, and stress than heterosexual women (18). A study by Alibudbud also states that LGBTQ+ Filipinos are excluded from the Filipino cultural practice that protects against increased anxiety (19). In addition, the WHO declared that common mental disorders are more prevalent among low-income households at about twice the rate compared to those of higher income levels (20). For instance, poor household income and poverty may be indicators of the development of depression, anxiety, and stress (21). Simultaneously, a larger family wealth might also relieve bad psychological experiences throughout childhood, which may affect students’ mental health after joining school (22).

Moreover, in terms of monthly household income levels, it was reported from past studies that students from low-income families are more at risk of encountering anxiety (15). Research also showed that participating in religion is inversely correlated with anxiety (15) and that pupils attending public or government schools were likelier to experience depression and anxiety (23, 24). Along with this, the study by Nadeem et al. revealed an inverse relationship between the religious conduct of students and psychological disorders, demonstrating that the increase in religious conduct decreases the risk of anxiety and stress among students (25). While public school pupils are more prone to experience sadness and anxiety, Deb S et al. suggests that children from either public or private schools who live in metro regions are more stressed than children who live in non-metro areas (26).

Studies have also found that genetics and family history significantly impact the early development of mental disorders, such as anxiety and depression (27). Among the biological influences, having a family history of mental illness is the most known (28). A systematic review of the genetic and environmental impacts on psychiatric comorbidity states that most patients diagnosed with a general anxiety disorder had the highest risk history, as indicated by their family’s psychiatric records (25). Moreover, a study assessing students’ mental health shows that a family history of mental illness and high anxiety scores are favorably associated with one another (29).

Regarding social factors, having a well-grounded social network and a stable and supportive relationship with their families can benefit students’ social and emotional well-being, lowering their likelihood of experiencing anxiety and depression during university (30). Therefore, a lack of support from family and university, negative connections with relatives, a lack of participation in social activities, extensive usage of social media, and belonging to ethnic and religious minority groups are associated with the mental well-being of college students (31). Similarly, social support has been found to be substantially and adversely related to student depression, anxiety, and stress, showing that the more support students receive, the less psychological distress they experience (32).

### 1.3. Objectives and significance

With the scarcity of literature concerning student mental health in the Philippines (11), this study determined the factors influencing the risk of anxiety, depression, and stress among senior high school students studying in Metro Manila during the COVID-19 pandemic. The factors explored that may influence anxiety, depression, and stress are sociodemographic characteristics, family history of a mental health disorder, and social support. The findings may contribute to understanding anxiety, depression, and stress among senior high school students and guide academic institutions in promoting students’ mental wellness and integrating safe spaces for mental health in their learning systems and environments as the Philippines recovers from the impact of the COVID-19 pandemic.

## 2. Methodology

This quantitative cross-sectional study used self-administered questionnaires, including the Depression, Anxiety, and Stress Scale-21 (DASS-21) and the Multidimensional Scale of Perceived Social Support (MSPSS) among senior high school students in Metro Manila. It determined the association between sociodemographic characteristics, family history of mental disorders, social support, and the risk for depression, anxiety, and stress among young adult senior high school students.

This study is part of a larger research about anxiety among high school students. It conformed to the Philippines’ Data Privacy Act and the National Ethical Guidelines for Health and Health-Related Research of the Philippines. In this regard, the study was ethically cleared at the De La Salle University, Manila – Integrated School. Informed consent was secured prior to data collection.

### 2.1. Population and sampling

The study recruited young adult senior high school students duly enrolled in public and private educational institutions within Metro Manila. Thus, students enrolled in schools outside Metro Manila were excluded. The sample size for this study was computed using G*Power 3, a statistical power analysis program for social and medical sciences (33). We set the sample size computation based on 10 predictors, an effect size of 0.15, an alpha error probability of 0.05, and a power of 0.95. The computed target sample size was 172 participants. The study employed convenience sampling in recruiting participants for the survey using Gmail and other social media platforms, such as Facebook, Messenger, and Twitter, which lasted 1 month. After the data collection period, 187 participants were eligible and had completed the study questionnaires.

### 2.2. Instrumentation

This study utilized a self-administered online survey. The survey questionnaires contained several items concerning possible risk factors for anxiety, depression, and stress, including sociodemographic characteristics, family mental health histories, and social factors.

The first section of the survey asked about the participants’ sociodemographic characteristics, such as age, assigned sex at birth, sexual orientation, monthly household income level, religious affiliation, and school type. The second section concentrated on the family mental health history of the respondents. Specifically, they were asked if they have a family member who has been professionally diagnosed with mental disorders.

The third section of the survey assessed depression, anxiety, and stress among the participants using the Depression, Anxiety, and Stress Scale-21 (DASS-21). The DASS-21 was developed by Lovibond and Lovibond and is the shortened version of the DASS-42, a self-report assessment used to assess negative emotions such as depression, anxiety, and stress (34). This scale is appropriate for clinical and non-clinical settings as a mental health screening test and to aid in diagnosing and outcome tracking. The DASS-21 has shown Cronbach’s alpha of 0.899 for the stress subscale, 0.861 for the anxiety subscale, and 0.863 for the depression subscale among Filipinos (13). Furthermore, the scale has demonstrated a substantial relationship with other depression and anxiety measures among Asians (35). In this study, the average scores for the depression, anxiety, and stress of each participant were calculated by summing up their scores on the corresponding subscales. Subsequently, these scores were used to classify the participants based on their risk for significant depression, anxiety, and stress. The study employed established cut-off scores of 10, 8, and 15, respectively, which had been previously utilized among Filipinos (13).

The fourth section focused on the senior high school students’ perceived social support. The adequacy of social support among the respondents was measured using the Multidimensional Scale of Perceived Social Support (MSPSS). It has three subscales considering different sources of social support, including family, friends, and significant others (36). An alpha coefficient of 0.847 has been observed for the overall scale regarding internal dependability (37). Additionally, a study on the internal reliability of MSPSS has been established with Filipino participants and indicated that Cronbach’s alpha of the scale is 0.89, which is good internal reliability (38). For this study, the level of social support from the participants’ family, friends, and significant others was calculated by summing up the scores for each subscale. This approach allowed the analysis of the specific level of each support source in the participants’ lives.

### 2.3. Data collection

The informed consent forms were located in the first part of the online questionnaire, where participants may indicate their voluntary involvement before completing the research instruments. Additionally, it contained comprehensive information on the study’s procedure, participant rights, data confidentiality, and the participant’s voluntary nature. In case the online survey causes participant discomfort, a list of mental health service providers was also provided. After obtaining the participants’ informed consent, they proceeded to answer the sociodemographic questionnaire, family history questionnaire, DASS-21, and MSPSS. The data collection period lasted 1 month (from February 2023 to March 2023) and reached the target sample size. Subsequently, the data was encoded in Microsoft Excel on a password-protected laptop. Numerical codes were utilized to protect the participants’ identities instead of identifying information in encoding their data.

### 2.4. Data analysis

Categorical data were summarized using frequency and percentages, while continuous data were summarized using standard deviation and means. Linear regression was performed to determine the factors associated with anxiety, depression, and stress. The dependent variables for the linear regression models were the scores in the subscales of the DASS-21. On the contrary, the predictors were sociodemographic characteristics, family history of mental disorders, and social support. The Beta coefficient and standard error were used to determine the factors influencing the risk for anxiety, depression, and stress. A *p*-value of <0.05 was considered significant. All statistical analyses were performed using the Statistical Package for the Social Sciences (SPSS).

## 3. Results

### 3.1. Sociodemographic characteristics, family history, social support, and risk for mental disorders of the participants

As shown in Table 1, the mean age of the participants is 18.15 (SD = 0.53). Most of them were females (*n* = 121, 64.71%), had an income of P43,828 or greater (*n* = 118, 63.11%), were Catholic (*n* = 150, 80.21%), and were studying at private schools (*n* = 133, 71.12%). More than a quarter of them also identified as LGBTQ+ (*n* = 47, 25.13%) and have a family history of mental disorders (*n* = 57, 30.48%). In this regard, the present study incidentally recruited a relatively large LGBTQ+ sample. In the Philippines, recent population-based surveys revealed that about 4 and 2% of males identified as bisexual and gay, while 10% of females aged 15 to 19 identifies as bisexual (39). Likewise, 8% of males and 5% females identified as transgender (39). Therefore, the relatively large proportion of LGBTQ+ individuals in this study is near the collective proportion of Filipino LGBTQ+ youth. Moreover, this proportion is similar to the rates found in other local studies involving the youth that collected sexuality and gender data (18, 19).

**Table 1:** Sociodemographic characteristics, family history, social support, and risk for mental disorders of the participants (*n* = 187).

	Mean/frequency	Std. deviation/ percentage
Sociodemographic characteristics		
Age	18.15	0.53
Sex assigned at birth		
Male	66	35.29
Female	121	64.71
LGBTQ+ identifying	47	25.13
Family monthly income in PhP		
Less than P10,957	19	10.16
P10,957 - P21,194	20	10.70
P21,194 - P43,828	30	16.04
P43,828 - P76,669	32	17.11
P76,669 - P131,484	35	18.72
P131,484 - P219,140	18	9.63
Greater than or equal to P219,140	33	17.65
Religion		
Catholic	150	80.21
Non-catholic	37	19.79
Type of school		
Public	54	28.88
Private	133	71.12
Family history of mental disorders	57	30.48
Social support		
Significant other	4.39	1.55
Family	3.25	1.79
Friends	4.55	1.36
Mental health level		
Depression score	11.10	4.73
Anxiety score	11.95	4.34
Stress score	11.55	4.13
Risk for poor mental health		
Depression	114	60.96
Anxiety	155	82.89
Stress	48	25.67

For social support, the participants indicated that they received the highest level of social support from their friends (mean = 4.55, SD = 1.36), followed by their significant other (mean = 4.39, SD = 1.55) and families (mean = 3.25, SD = 1.79). For mental health, the participants indicated the highest score on the anxiety subscale (mean = 11.95, SD = 4.34), followed by the stress (mean = 11.55, SD = 4.13) and depression (mean = 11.10, SD = 4.73) subscales. Likewise, three out of five participants were also found to have a significant risk for depression (*n* = 114.00, 60.96%), while four out of five participants were at significant risk for anxiety (*n* = 155.00, 82.89%). Finally, one out of four participants was at risk for significant stress (*n* = 48.00, 25.67%).

### 3.2. Model summary of the regression models for depression, anxiety, and stress

Table 2 shows the three models used to predict the association between sociodemographic characteristics, family history of mental disorders, social support, and the risk for depression, anxiety, and stress among the participants. The variables of model 1 showed collective significance in predicting the risk for depression, *F* (17, 169) = 5.050, *p* < 0.001. Based on its adjusted R Square, 17.9% of the variance of depression among the participants could be attributed to the predictors of Model 1. Similarly, the variables of model 2 showed collective significance in predicting the risk for anxiety among the participants, *F* (17, 169) = 3.306, *p* = 0.001. Based on its adjusted R Square, 11.0% of the variance of anxiety could be attributed to its predictors. Lastly, the variables of model 3 showed collective significance in predicting the risk of anxiety among the participants, *F* (17, 169) = 3.255, *p* = 0.001. Based on its adjusted R Square, 10.8% of the variance of significant stress among the participants could be attributed to its predictors.

**Table 2:** Model summary of the regression models for depression, anxiety, and stress.

Model	Dependent variable	*R*	*R* square	Adjusted *R* square	Total df	*F*	Sig.
1	Depression	0.472	0.223	0.179	186	5.050	<0.001
2	Anxiety	0.398	0.158	0.110	186	3.306	0.001
3	Stress	0.395	0.156	0.108	186	3.255	0.001

### 3.3. Model summary of the regression models for depression, anxiety, and stress

Table 3 shows the results of the regression models for depression, anxiety, and stress among the participants. It shows that higher social support from the family was negatively associated with depression, anxiety, and stress scores among the participants (*p* < 0.05). In addition, female sex at birth, compared to male sex, was positively associated with anxiety and stress scores among the participants (*p* < 0.05). Likewise, having a family history of depression positively associated with depression, anxiety, and stress (*p* < 0.05). On the contrary, the findings also revealed that the risk for significant levels of depression, anxiety, and stress among the participants has no statistically significant association with age, LGBTQ+ identity, family monthly income, religion, type of school, and social support from significant others and friends.

**Table 3:** Association between sociodemographic characteristics, family history of mental disorders, social support, and the risk for depression, anxiety, and stress among the participants.

	Depression			Anxiety			Stress		
	*B*	Std. error	*p*	*B*	Std. error	*p*	*B*	Std. error	*p*
Socio-demographic characteristics									
Age	−0.349	0.613	0.57	0.498	0.585	0.396	−0.285	0.558	0.610
Sex at birth									
Male	Referent								
Female	0.598	0.673	0.375	1.967^*^	0.642	0.003	1.317^*^	0.612	0.033
LGBTQ+ identifying	−0.375	0.761	0.623	−0.378	0.727	0.604	−1.057	0.693	0.129
Income	0.184	0.190	0.334	0.021	0.182	0.909	−0.052	0.173	0.763
Religion									
Catholic	Referent								
Non-catholic	−0.450	0.820	0.584	−1.093	0.783	0.164	−0.412	0.746	0.582
School type									
Public	Referent								
Private	−0.647	0.801	0.420	−0.834	0.764	0.277	−0.073	0.729	0.920
Family history of mental disorders	2.295^*^	0.720	0.002	1.809^*^	0.688	0.009	2.508^*^	0.656	<0.001
Social support									
Significant other	0.075	0.246	0.761	−0.016	0.235	0.947	−0.051	0.224	0.820
Family	−1.048^*^	0.194	0.000	−0.597^*^	0.185	0.002	−0.548^*^	0.177	0.002
Friends	0.065	0.269	0.810	−0.044	0.257	0.863	0.177	0.245	0.469

^*^p < 0.05.

Overall, the findings suggest that social support from one’s family may be protective factors for higher levels of depression, anxiety, and stress among the participants. On the other hand, having female sex may be a risk factor for higher anxiety and stress. Similarly, a family history of mental disorders may be a risk factor for higher levels of depression, anxiety, and stress.

## 4. Discussion

The findings revealed that four-fifths of senior high school students from Metro Manila are at significant risk for anxiety, three-fifths of the participants are at risk for depression, and one-fourth are at risk for stress. Compared to a study conducted by Alibudbud among young Filipino students, the rate of students in the current study who are at risk for anxiety and/or depression is twice as high (19). Another study has shown an increasing trend in the risk of mental health problems among the student population in the Philippines during the COVID-19 pandemic (40). The present study’s findings supported this increasing trend, showing certain social factors that may influence anxiety.

The present study’s results also suggest that social support from an individual’s family and significant others may be protective factors for significant depression among the participants. These results are similar to the findings of Mariani et al. which found that good social support reduces one’s risk of anxiety and depression (41). In addition, Billote et al. also mentioned that opening up to a friend relieves some of the participants (42). Moreover, having a supportive social network may influence students’ social and emotional well-being and subsequently lower their probability of having anxiety and depression (31), which reaffirms the present study’s findings. With that, it may be inferred that these social factors significantly influence senior high school students’ risk for depression, anxiety, and stress.

Furthermore, the findings showed that female senior high school students were more likely to experience significant anxiety and stress than male students. This result is consistent with previous studies suggesting that a person’s sex influences anxiety among senior high school students (14, 43). Moreover, it was said that women had a high prevalence rate of experiencing stress, anxiety, and other mental health issues (44, 45). Furthermore, according to Thawabieh and Qaisy, female students further identified stress-related issues such as depression, examination stress, and low self-esteem (46). Therefore, sex at birth may be considered a factor influencing anxiety and stress among senior high school students. However, further investigation is necessary to thoroughly comprehend how a person’s sex affects their mental health in the Philippines.

Similar to previous studies, a family history of mental illness increases students’ likelihood of emotional distress (47–50). In addition, according to Ghodasara et al. having a family history of mental health disorders increases the risk of depression (29). This result may be explained by hereditary factors and the difficulties of caring for a mentally ill family member (47). Thus, it may be deduced that the risk for significant stress levels in senior high school students is influenced by their family history of mental disorders.

In contrast to previous research, the findings suggest that age, LGBTQ+ identity, family monthly income, religion, type of school, and social support from friends have no statistically significant correlation with the risk of significant levels of depression, anxiety, and stress among the participants (14, 15, 21, 22). This lack of statistical association can be further explored in future studies.

### 4.1. Limitations

This study is the first to explore the factors influencing anxiety among students, specifically senior high school students in Metro Manila. However, its potential limitations should be noted. First, the participants in this study may not cover a diverse population. Due to the sampling approach, it may have limited generalizability. Moreover, compared to a previously conducted representative population survey (39), our sample has more females than that seen from the general population. Therefore, future research can utilize non-probability sampling methods for a better representation and generalizability. Second, a limited number of mental health factors influencing anxiety, depression, and stress were explored in this study. Likewise, COVID-19 pandemic-related factors, such as exposure to lockdowns and isolation measures, were not explored in the present study. Hence, future studies can explore other factors, such as potential genetic factors, life stressors, and COVID-19 pandemic experiences.

Furthermore, qualitative studies focusing on the depth, meaning, and other issues of those suffering from anxiety, depression, and stress can be conducted in the future. Lastly, as with most cross-sectional designs, this study cannot adequately evaluate the causal relationship between variables. Thus, future longitudinal research investigating causal relationships may be conducted.

## 5. Conclusion

The study determined several factors influencing depression, anxiety, and stress among senior high school students in Metro Manila. These factors include one’s sex at birth, family history of mental health issues, and social support from their family and significant others. While the study has certain limitations, it can be recommended that social support be strengthened among senior high school students to improve their mental health. In addition, students at risk for poor mental health, including females and those with a family history of mental disorders, may need additional support in school mental health programs. Nevertheless, further research is needed to fully understand mental health among Filipino senior high school students.

## Data availability statement

The raw data supporting the conclusions of this article will be made available by the authors, without undue reservation.

## Ethics statement

The studies involving humans were approved by De La Salle University, Manila – Integrated School. The studies were conducted in accordance with the local legislation and institutional requirements. The participants provided their written informed consent to participate in this study.

## Author contributions

IS, AC, KC, and JM had substantial contributions to the design, drafting, revision, acquisition, interpretation, and final approval of the data and work. RA had substantial contributions to the design, drafting, revision, data analysis, and final approval of the data and work. All authors contributed to the article and approved the submitted version.

## Conflict of interest

The authors declare that the research was conducted in the absence of any commercial or financial relationships that could be construed as a potential conflict of interest.

## Publisher’s note

All claims expressed in this article are solely those of the authors and do not necessarily represent those of their affiliated organizations, or those of the publisher, the editors and the reviewers. Any product that may be evaluated in this article, or claim that may be made by its manufacturer, is not guaranteed or endorsed by the publisher.
